# Calcium dobesilate reduces SARS-CoV-2 entry into endothelial cells by inhibiting virus binding to heparan sulfate

**DOI:** 10.1038/s41598-022-20973-3

**Published:** 2022-10-07

**Authors:** Yulia Kiyan, Anna Schultalbers, Ekaterina Chernobrivaia, Sergey Tkachuk, Song Rong, Nelli Shushakova, Hermann Haller

**Affiliations:** 1 Department of Nephrology, Hannover Medical School, Carl-Neuberg-Str. 1, 30625 Hannover, Germany; 2 Mount Desert Biological Laboratory MDIBL, Bar Harbor, USA; 3 Phenos GmbH, Hannover, Germany

**Keywords:** Cell biology, Mechanisms of disease

## Abstract

Recent reports demonstrate that SARS-CoV-2 utilizes cell surface heparan sulfate as an attachment factor to facilitate the initial interaction with host cells. Heparan sulfate interacts with the receptor binding domain of SARS-CoV-2 spike glycoprotein, and blocking this interaction can decrease cell infection. We and others reported recently that the family of compounds of 2,5-dihydroxyphenylic acid interferes with the binding of the positively charged groove in growth factor molecules to negatively charged cell surface heparan sulfate. We hypothesized that Calcium Dobesilate (CaD)—calcium salt of 2,5-dihydroxyphenylic acid—may also interfere with the binding of SARS-CoV-2 spike protein to heparan sulfate. Using lentiviral SARS-CoV-2 spike protein pseudotyped particles we show that CaD could significantly reduce pseudovirus uptake into endothelial cells. On the contrary, CaD did not affect cell infection with VSVG-expressing lentivirus. CaD could also prevent retention of SARS-CoV-2 spike protein in ex vivo perfused mouse kidney. Using microfluidic culture of endothelial cells under flow, we show that CaD prevents spike protein interaction with heparan sulfate glycocalyx. Since CaD has no adverse side effects and is approved in humans for other medical indications, our findings can rapidly translate into clinical studies.

## Introduction

Infection with severe acute respiratory syndrome coronavirus 2 (SARS-CoV-2) is in most patients limited to bronchoalveolar epithelial cells and associated with local bronchopulmonary symptoms^1–3^⁠. This form of infection is self-limiting and causes no serious threat to the infected person. However, in 20% of patients, infection with SARS-CoV-2 leads to life-threatening respiratory, cardiac, renal, and cerebral injury^1,3^⁠. The underlying mechanisms of this severe form of the disease are not fully understood. The clinical picture in these patients resembles the systemic inflammatory response syndrome (SIRS) with a strong microvascular component^4–7^⁠. It seems that an inflammatory endothelial response that may involve the coagulation cascade and/or the complement pathway plays a vital role^8,9^⁠. Endothelial activation results into elevated levels of pro-inflammatory cytokines—interleukin-1, interleukin-6 (IL-6) and tumor necrosis factor-α (TNFα), chemokines and antithrombotic factors—von Willebrand factor and factor VIII in these critically endangered patients^10^⁠. Higher levels of acute phase reactants (IL-6, C-reactive protein, and D-dimer) are also associated with severe SARS-CoV-2 infection. Therefore, it is reasonable to assume that endothelial dysfunction contributes to COVID-19-associated vascular inflammation. Pro-inflammatory cytokines, elevated in patients with COVID-19, induce the loss of the normal antithrombotic and anti-inflammatory functions of endothelial cells, leading to coagulation dysregulation, complement and platelet activation, and leukocyte recruitment in the microvasculature^9^. Furthermore, recent report suggested that proteolytically released S1 domain of SARS-CoV-2 spike protein induces endothelial damage, vascular permeability and expression of PAI-1 and VEGF by lung microvascular endothelial cells^11,12^⁠.

These microvascular complications of SARS-CoV-2 infection are most likely responsible for the high rate of morbidity and mortality of infected patients^13,14^. There is still controversy existing whether SARS-CoV-2 infects the endothelium directly. Multiple reports demonstrate infection of endothelium both, in vivo and in vitro^15–18^⁠. Whereas other investigation find no evidence for endothelial infection^19,20^⁠. Organ-specific and regulated expression of ACE2 is important for cell infection with SARS-CoV-2^21^⁠. Expression of ACE2 by endothelial cells has been documented^22^⁠. Some organ-specific endothelial cells such as from brain show high ACE2 levels even in normal conditions^23^⁠. Another important factor that could potentially explain this controversy is the abundance of endothelial glycocalyx both in vivo and in vitro experimental systems^24^⁠.

The glycocalyx is a complex mixture of glycans and glycoproteins covering the endothelial cell surface. Viruses and other infectious organisms must transgress the glycocalyx to engage receptors that mediate viral entry into host cells. Many viral pathogens have evolved to utilize glycans as attachment factors, which facilitate the initial interaction with host cells, including alpha-, beta- and gammaherpesviruses^25–27^⁠⁠, influenza virus, immunodeficiency virus, and different coronaviruses (SARS-CoV-1 and MERS-CoV)^28,29^⁠. Several viruses interact with sialic acids located on the ends of glycans found in glycolipids and glycoproteins^30^⁠. Other viruses interact with heparan sulfate (HS)^31,32^⁠. This initial attachment of the virus to HS can lead to the engagement of protein receptors on the host plasma membrane, such as ACE2, that facilitate membrane fusion or engulfment and internalization.

Esko and his group have recently shown that the ectodomain of the SARS-CoV-2 spike protein interacts with cell surface HS through the receptor-binding domain. Surface binding to cells and virus uptake requires the engagement of both cellular HS and ACE2, suggesting that HS acts as a co-receptor priming the virus envelope for ACE2 interaction^33^⁠. These findings identify cellular HS as a necessary co-factor for SARS-CoV-2 infection and emphasize the potential for targeting spike protein–HS interactions to attenuate virus infection⁠. Further reports confirmed this observation^34^⁠. Yue and colleagues^35^⁠ investigated the role of heparin and HS sulfation for the binding of spike protein in details. They have also demonstrated that spike protein of highly infective G614 SARS-CoV-2 variant has higher binding to heparin and HS. Application of heparin in COVID-19 patients exerted beneficial effects via anti-coagulant and non-anticoagulant mechanisms^36,37^⁠. On the contrary, Targosz-Korezka and colleagues have reported recently that HS glycocalyx shields endothelial cell surface and prevents SARS-CoV-2 interaction with ACE2 receptor^38^⁠. Even more complexity was hypothesized by Buqaileh et al., who suggested that primary ciliary pockets—membranous invagination around the primary ciliary of endothelial cells—can serve as specific entry points for SARS-CoV-2 as ACE2 was concentrated at those area^39^⁠. When interpreting experimental data on the role of the glycocalyx in the virus entry mechanisms, it should be kept in mind that correct physiological models must be applied.

We and others recently identified a family of small molecules, 2,5-dihydroxyphenylic acid (2,5-DHPA), which interfere with the binding of the positively charged groove in growth factors to negatively charged HS^40,41^⁠. We hypothesized that 2,5-DHPA might also interfere with the binding of SARS-CoV-2-HS binding.

Calcium dobesilate (CaD)—calcium salt of 2,5-dihydroxyphenylic acid CaD is a vasoprotective drug that demonstrated beneficial effects on vascular leakage and inflammation in diabetic retinopathy^42–44^⁠, diabetic kidney damage^45^⁠, and venous insufficiency^46^⁠. Structure of CaD is shown in Supplementary Fig. 1A. This small molecule has been used for the treatment of various vascular disorders for the last few decades. CaD can improve microvascular abnormalities by regulating platelet activity and capillary permeability^47^⁠. However, the mechanism of action of CaD is not yet fully understood. 2,5-DHPA compound class interferes with growth factor signaling. For example, CaD binds to the heparin-binding domain of Fibroblast Growth Factor-1^40,48^⁠. We have recently shown that CaD could function similarly as a VEGF antagonist without adverse side effects^41^⁠. Recent meta-analyses further demonstrated the clinical safety of CaD^46,49–52^⁠.

We have developed a lentiviral expression system of luciferase- and GFP-expressing SARS-CoV-2 spike pseudotyped viral particles and studied uptake in cultured microvascular endothelial cells. We showed that CaD interferes with the binding of the spike protein to heparan sulfate and reduces pseudovirus uptake into endothelial cells. 2,5-DHPA (and possible derivatives) are potent inhibitors of SARS-CoV-2-HS binding and have the potential to prevent viral uptake and endothelial dysfunction in infected patients. Since CaD has been approved in humans for other medical indications, our findings can rapidly translate into clinical studies.

## Results

### CaD prevents binding of SARS-CoV-2 spike protein to the endothelial cells

**Figure 1:**
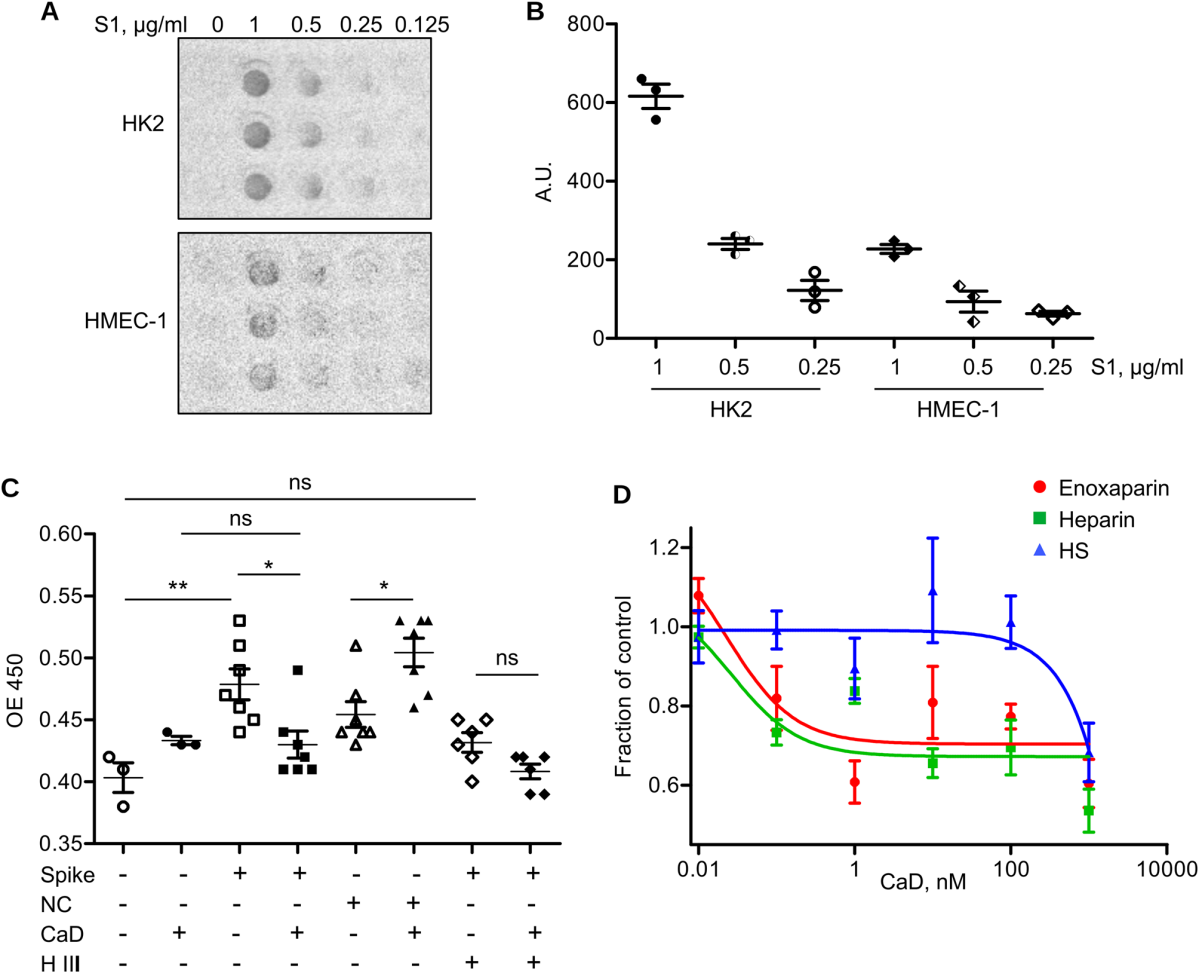
Endothelial and epithelial cells bind recombinant SARS-CoV-2 spike protein. (**A**) Binding of recombinant S1 subunit of SARS-CoV-2 spike protein by HK2 cells and HMEC-1 was assessed by dot blot using anti-spike protein antibody. (**B**) Quantification of the dot blot shown in (**A**). (**C**) Binding of His-tagged recombinant full length SARS-CoV-2 spike protein by HUVECs was assessed by cell-based ELISA using anti-His antibody. CaD (100 µM) was added together with spike protein. SARS-CoV-2 nucleocapsid protein (NC) was used to show the specificity of CaD towards spike protein. Cell were pre-treated with Heparinase III (H III) as indicated to shed HS from the cell surface. (**D**) Inhibition of spike protein binding to heparin, enoxaparin and HS by CaD. Recombinant spike protein was added to the plates coated with heparin, enoxaparin or HS in the presence of CaD. Binding was assessed using anti-His antibody.

First, we assessed whether kidney epithelial (HK-2) and human microvascular endothelial cells (HMEC-1) can bind recombinant SARS-CoV-2 spike protein by dot blot assay (Fig. 1A,B). S1 subunit of spike protein has been used. Spike protein binding detected using anti-spike antibody was observed in both, endothelial and epithelial cells though the former demonstrated lower binding. To get more quantitative data and test possible effects of CaD, cell-based ELISA has been performed using His-tagged SARS-CoV-2 spike protein. His-Tagged SARS-CoV-2 nucleocapsid protein was used as negative control to confirm that binding was specific for spike protein and not His-tag mediated (Fig. 1C). Using anti-His antibody we found that SARS-CoV-2 spike was bound to human umbilical vein endothelial cells (HUVECs). Adding CaD decreased spike binding to the cell surface. Nucleocapsid protein was bound to the HUVECs to a lesser degree and its interaction was not inhibited but rather increased by CaD. Cell treatment with heparinase III that sheds HS glycocalyx from the cell surface decreased spike S1 binding and abrogated effects of CaD. HS antibody was used in cell based ELISA to make sure that Heparinase III decreased the content of HS on the cell surface (Supplementary Fig. 1B). This data suggested that CaD interferes with the binding of spike protein with cell surface HS.

To gain better insight in the mechanisms of CaD inhibition of spike protein binding to HS, in vitro binding assay was performed. Spike protein was incubated in the plates coated with low molecular weight heparin enoxaparin, heparin and HS from bovine kidney^33^⁠⁠ in the presence of different concentrations of CaD (Fig. 1D). CaD significantly inhibited binding of spike to heparin and enoxaparin. Spike protein binding to the HS preparation that we used in our experiments in vitro was significantly less in comparison to heparin and enoxaparin (Supplementary Fig. 1C). However, higher concentrations of CaD were needed to inhibit the binding with IC50 0.023, 0.026 and 507 nM for enoxaparin, heparin and HE, respectively. HS represents a mixture of chains of various molecular weight and significantly less sulfated than heparin. Due to difference in sulfation pattern and molecule charge, it is difficult to exclude the possibility that immobilization of HS on the plate is different and observed effects are due to less efficient immobilization of HS. This data suggests that sulfation of glycosoaminoglycans is an important factor for spike protein binding and inhibition of the binding by CaD.

### CaD prevents endothelial cell infection with spike protein pseudotyped lentivirus

We developed pseudotyped viral particles expressing the spike protein of the SARS-CoV-2 virus on the lentiviral core. Expression of Gaussia luciferase (Gluc) or GFP was used as reporter to quantify cell infection rate. The expression of SARS-CoV-2 spike protein in pseudoviral particles has been verified by Western blotting (Supplementary Fig. 1D). Observed molecular weight corresponded the predicted glycosylated form of spike protein of 144–175 kDa.

**Figure 2:**
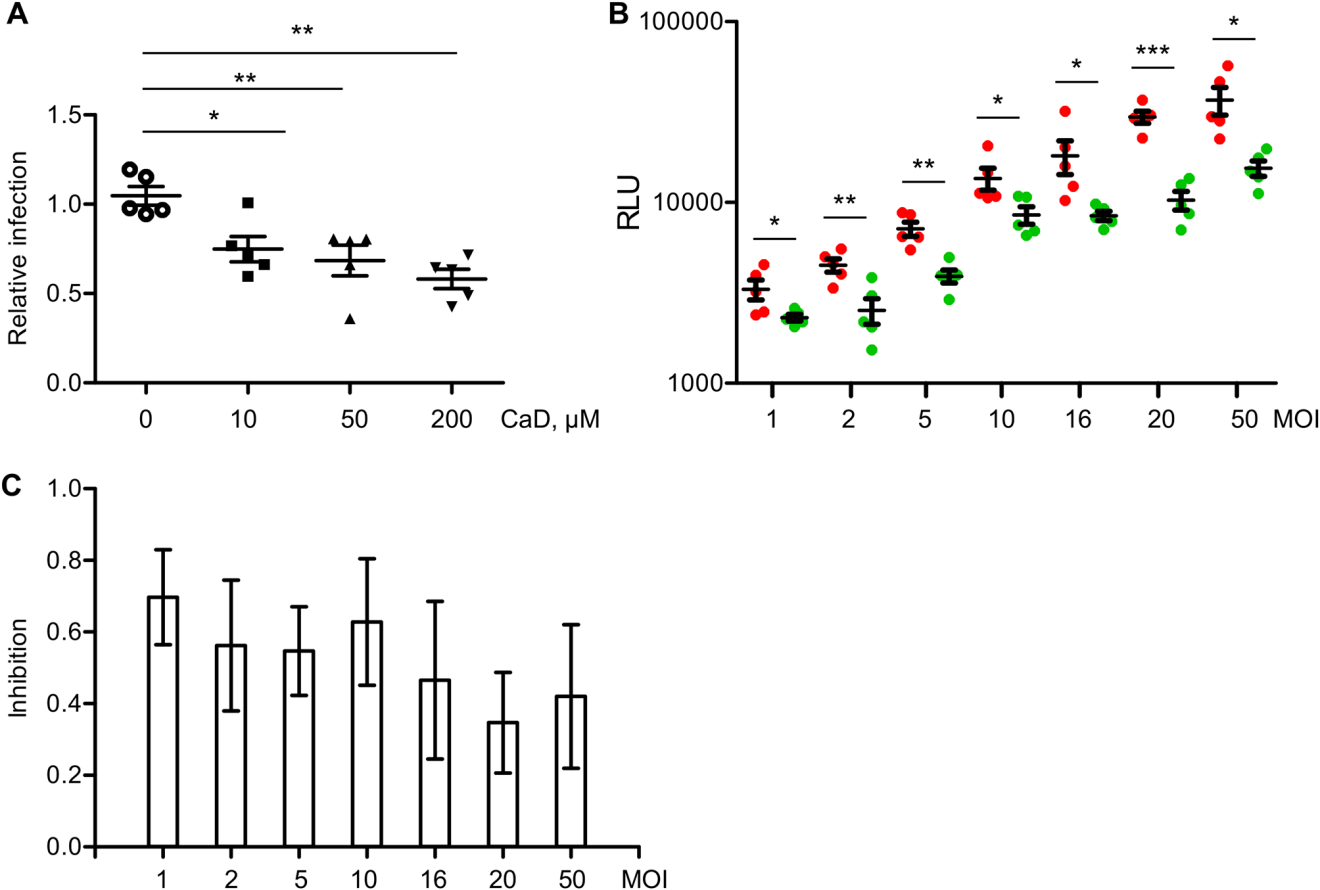
CaD inhibits endothelial cell infection with SARS-CoV-2 spike-pseudotyped lentivirus. (**A**) HUVECs were infected with spike-pseudotyped lentivirus expressing Gaussia luciferase as a reporter in the presence of indicated CaD concentrations. Virus was added to the cells for 2 h and then cells were washed and fresh medium was given. Activity of luciferase in conditioned medium was measured 24 h after cell infection. (**B**) HMEC-1 endothelial cells were infected with spike protein pseudotyped virus at MOI from 1 to 50 in the presence or absence of 10 µM CaD. Infection and measurement of luciferase activity was performed as in (**A**). (**C**) Folds of inhibition of cell infection by CaD at different MOI calculated from data shown in (**B**).

To gain quantitative data on the effects of CaD, HUVECs infection with spike protein pseudotyped lentivirus has been performed. Pseudovirus was added to the cells for 2 h in the presence or absence of CaD. Then, virus-containing medium was removed, cells were washed with warm medium to remove inbound virus particles, and fresh medium was added to the cells. CaD inhibited cell infection that was determined by measuring Gluc activity in cell conditioned medium (Fig. 2A). Then, we infected HMEC-1 cells with spike protein pseudotyped lentivirus at multiplicity of infection (MOI) varying from 1 to 50. Inhibition of the cell infection by CaD was observed at all MOI (Fig. 2B,C) showing a trend to more efficient inhibition at higher MOI values.

**Figure 3:**
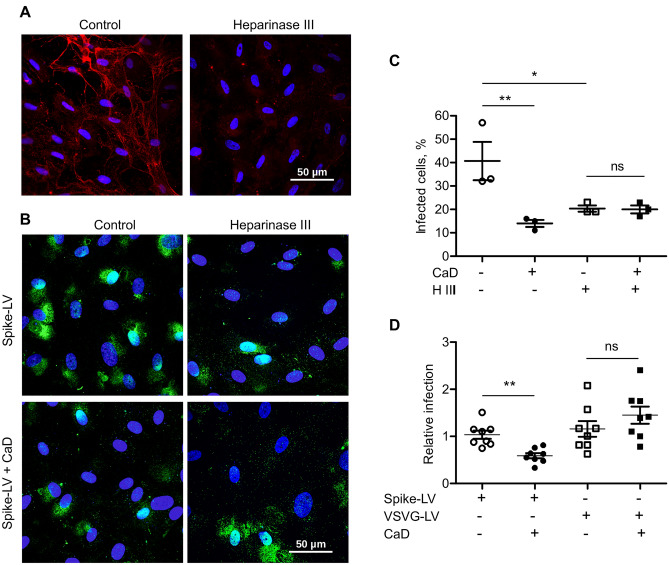
CaD inhibits interaction of SARS-CoV-2 spike protein with HS. (**A**) HUVECs were treated with Heparinase III for 1 h and then fixed and stained with heparan sulfate antibody (red) and DAPI. Intracellular HS staining is still present, whereas extracellular fibrous HS staining was removed by Heparinase III treatment. (**B**) Heparinase III treatment was performed for 1 h at 37 °C prior cell infection. HUVECs were infected with spike-pseudotyped lentivirus (Spike-LV) expressing GFP as a reporter in the presence or absence of 10 µM CaD. 24 h after infection cells were fixed and expression of GFP was visualized by microscopy. (**C**) The number of pseudovirus-infected GFP positive cells was Quantified using ImageJ. (**D**) TaqMan RT-PCR was performed to quantify GFP mRNA expression in HUVEC infected with Spike-pseudovirus and VSVG protein lentivirus (VSVG-LV) in the presence of CaD.

Then, to gain insight into the role of HS in the CaD mediated inhibition, we treated HUVECs with Heparinase III for 1 h prior infection. Staining with HS antibody confirmed essential decrease of cell surface HS after Heparinase III treatment (Fig. 3A). Then, cells were infected with GFP-reporter spike protein pseudotyped lentivirus for 2 h in the presence of CaD. Then virus containing medium was removed. After 48 h post infection the number of GFP-expressing cells was quantified (Fig. 3B,C). We observed decreased cell infection with spike pseudovirus in the presence of CaD and after removal of cell surface HS by Heparinase III (Fig. 3C). On the contrary, CaD demonstrated no inhibitory effect on cell infection with VSVG-lentivirus (Fig. 3D).

**Figure 4:**
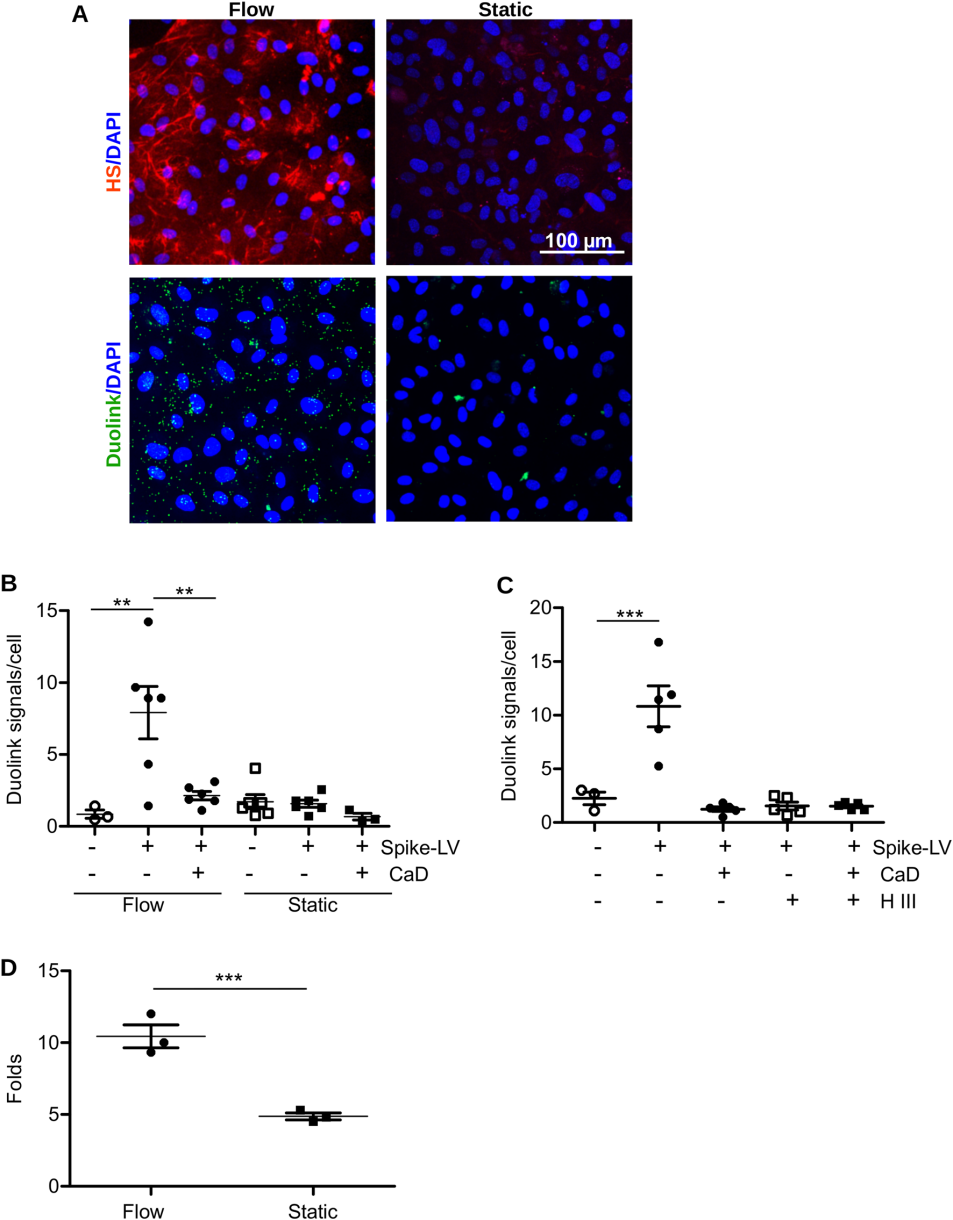
SARS-CoV-2 spike protein interaction with HS is blocked by CaD. Duolink assay was performed on HUVECs grown under flow or static conditions for 5 days. (**A**) Staining of HS glycocalyx was performed using anti-HS antibody. Lower panels show duolink signals in green. DAPI was used as nuclear stain. (**B**) Quantification of the duolink signals number/cell using ImageJ software. (**C**) HUVECs grown under flow conditions for 5 days were treated with Heparinase III for 1 h prior to the addition of the spike pseudotyped lentivirus. (**D**) GFP expression after HUVEC infection under static and flow conditions was assessed by RT-PCR.

It is well recognized that endothelial cells cultured under standard cell culture conditions express low level of HS glycocalyx. Shear stress stimulation is necessary to induce expression of HS and formation of 3D luminal glycocalyx structure that resembles in vivo glycocalyx layer^24^⁠. We applied custom microfluidic cell culture system which was recently developed by our group^53^⁠ to assess the role of HS in cell infection with spike pseudovirus under more physiological conditions. HUVECs were seeded in the microfluidic chips and allowed to grow under medium flow conditions for 5 days. Then, spike protein pseudotyped lentivirus was added and cells were fixed after 30 min of incubation. We applied Duolink in situ proximity ligation assay to visualize direct interaction of spike protein with HS. 10e4 antibody were used to visualize HS. HUVECs incubated under flow conditions demonstrated higher expression of HS in comparison to cells grown under static conditions (Fig. 4A). Accordingly, higher number of direct interaction was detected using Duolink proximity ligation assay performed using anti-spike and anti-heparan sulfate antibody (Fig. 4A). Strong inhibition of this interaction by CaD was confirmed by quantification of the duolink positive signals number (Fig. 4B). Duolink assay was also performed in cells grown under flow conditions and then treated with Heparinase III before addition of pseudotyped virus (Fig. 4C). Control staining of cell HS after the treatment with Heparinase III is shown in Supplementary Fig. 2A. In agreement with the previous data, HS removal decreased the virus binding to the cell surface. Accordingly, HUVECs grown under flow conditions were more prone to the infection with spike protein pseudotyped virus as was quantified by the TaqMan RT-PCR 48 h after cell infection (Fig. 4D).

### CaD effects on cell infection are not mediated by cell toxicity or inhibition of endocytosis

**Figure 5:**
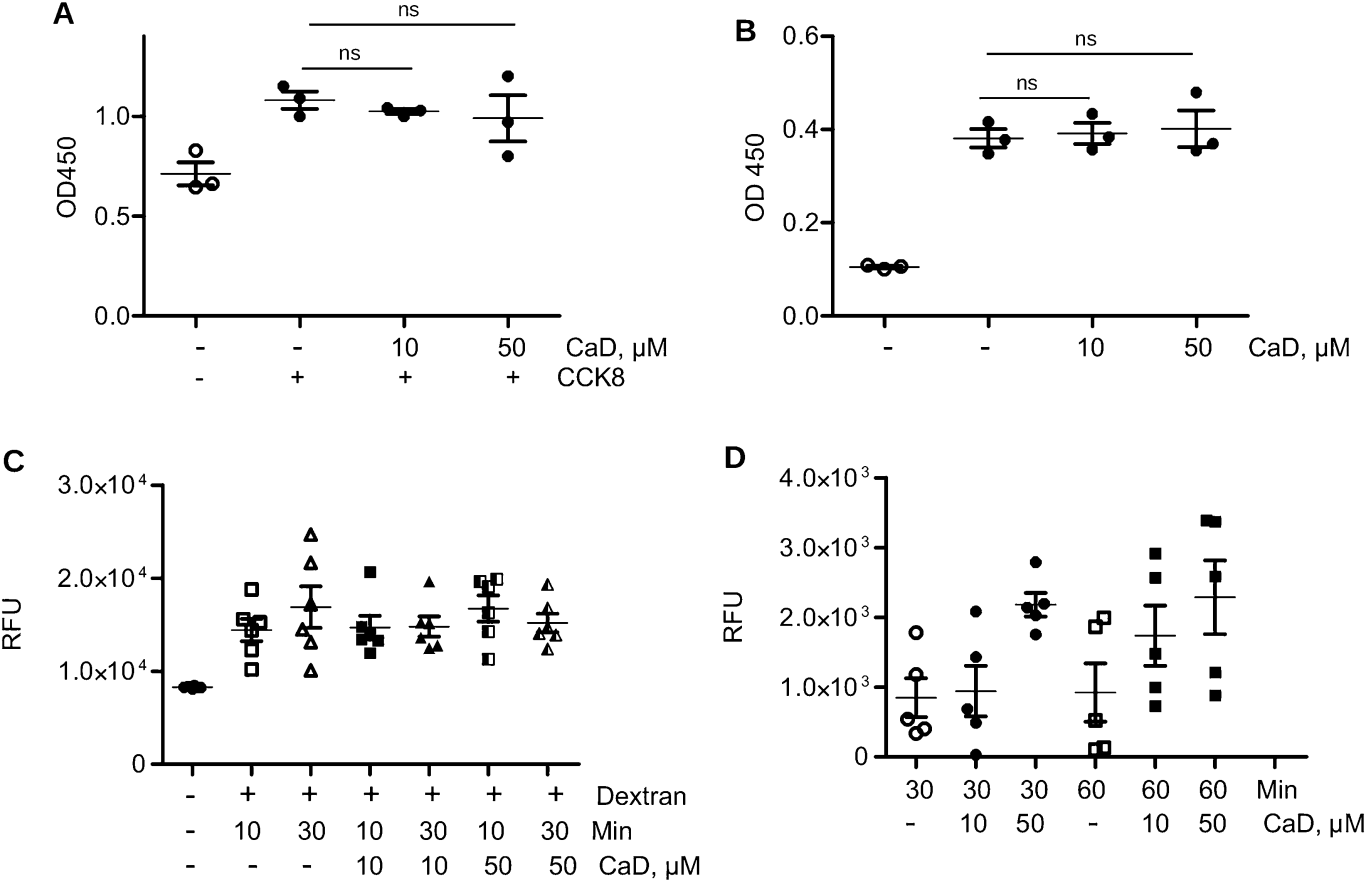
CaD does not cause cell toxicity. (**A**) HMEC-1 were treated with CaD as for cell infection described in Methods. Toxicity was determined by CCK-8 kit 24 h after CaD treatment. Statistical significance was calculated against control without CaD. (**B**) Proliferation of endothelial cells in the presence of indicated concentrations of CaD was assessed using the BrdU incorporation assay (Roche). Statistical significance was calculated against control without CaD. (**C**) Cells were incubated with FITC-Dextran for 10 min and 30 min in the presence of indicated concentrations of CaD, then washed, and fluorescence was measured as described in the methods. Statistical significance was calculated against control without CaD. (**D**) Phagocytosis of FITC-labelled *E. coli* was assessed in endothelial cells after incubation for 30 and 60 min. CaD was added as indicated.

We next wanted to exclude possible indirect effects of CaD which are not mediated by spike protein/HS interaction. Several possible mechanisms were investigated. First, we assessed CaD dependent cell toxicity using the Cell Counting Kit 8 (CCK8). Our data confirmed that CaD did not induce any cell toxicity under our experimental conditions (Fig. 5A). Second, we used BrdU incorporation assay to assess cell proliferation and observed that there were no effects of CaD on cell proliferation under these experimental conditions (Fig. 5B).

Further, to exclude possible effects of CaD on the virus internalization processes, we investigated pinocytosis and phagocytosis. We employed FITC-Dextran and FITC-labelled *E. coli*, respectively. CaD exerted no significant effects on these processes (Fig. 5C,D). These data support a specific effect of CaD on virus binding to HS in the early stages of virus attachment and receptor interaction and rule out cytotoxicity of CaD and direct interference of CaD with the internalization process.

### CaD prevents spike protein binding in ex vivo perfused mouse kidney

**Figure 6:**
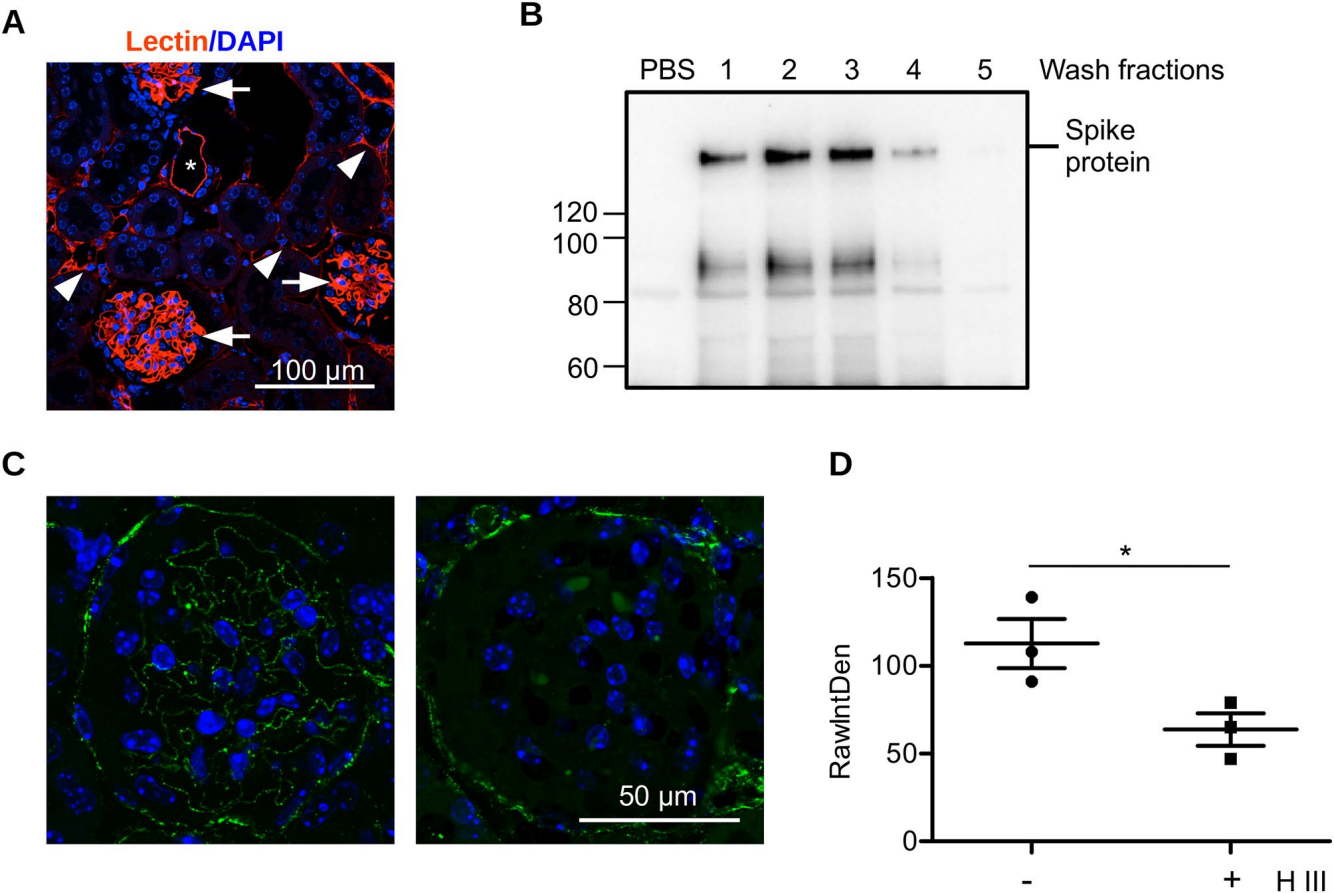
Ex vivo kidney perfusion system. (**A**) Mouse kidney were perfused ex vivo with lectin. Perfusion of glomeruli (arrows), artery (asterisks), and peritubular capillary (arrowheads) was achieved. (**B**) Mouse kidney were perfused with 15 µg/ml of recombinant SARS-CoV-2 spike protein in PBS for 30 min. Then, the kidney were washed with PBS and perfusate was collected in fraction (1–5). Then, kidney were frozen in liquid nitrogen for biochemical assay of spike protein and fixed for immunohistochemistry. (**C**) Paraffin-embedded kidney slices of the perfused kidney (left) and the kidney perfused with Heparinase III (right) stained for HS and DAPI. (**D**) Quantification of HS expression in the kidney glomeruli without and with Heparinase III treatment.

We applied next ex vivo mouse kidney perfusion to test SARS-CoV-2 spike protein binding in the kidney. Isolated mouse kidneys were connected to the peristaltic pump and perfused with PBS solution containing spike protein in the presence or absence of CaD. To confirm effective tissue perfusion and preservation of the endothelial glycocalyx, Sambucus Nigra lectin was added to the PBS during perfusion. Figure 6A shows strong lectin labeling of endothelial cells in the glomeruli, peritubular capillary and large vessels. After establishing the perfusion conditions, recombinant spike protein was added and perfusion was performed during 30 min. To remove unbound spike protein, kidneys were then perfused with PBS for further 30 min. PBS from tissue wash was collected in fractions and analyzed for the presence of spike protein. Figure 6B demonstrates that unbound spike protein was completely removed. Further, we performed pre-treatment of the kidney with Heparinase III. The extent of HS cleavage was assessed by staining the kidney slices with anti-HS antibody. Figure 6C shows staining of the kidney glomerulus after treatment with Heparinase III. Significant decrease of HS content in capillary was achieved whereas Bowmann capsule is stained. Quantification of the HS staining in kidney glomeruli was performed using ImageJ software (Fig. 6D). The detection of bound spike protein in the tissue was performed by western blotting analysis and immunohistochemistry.

**Figure 7:**
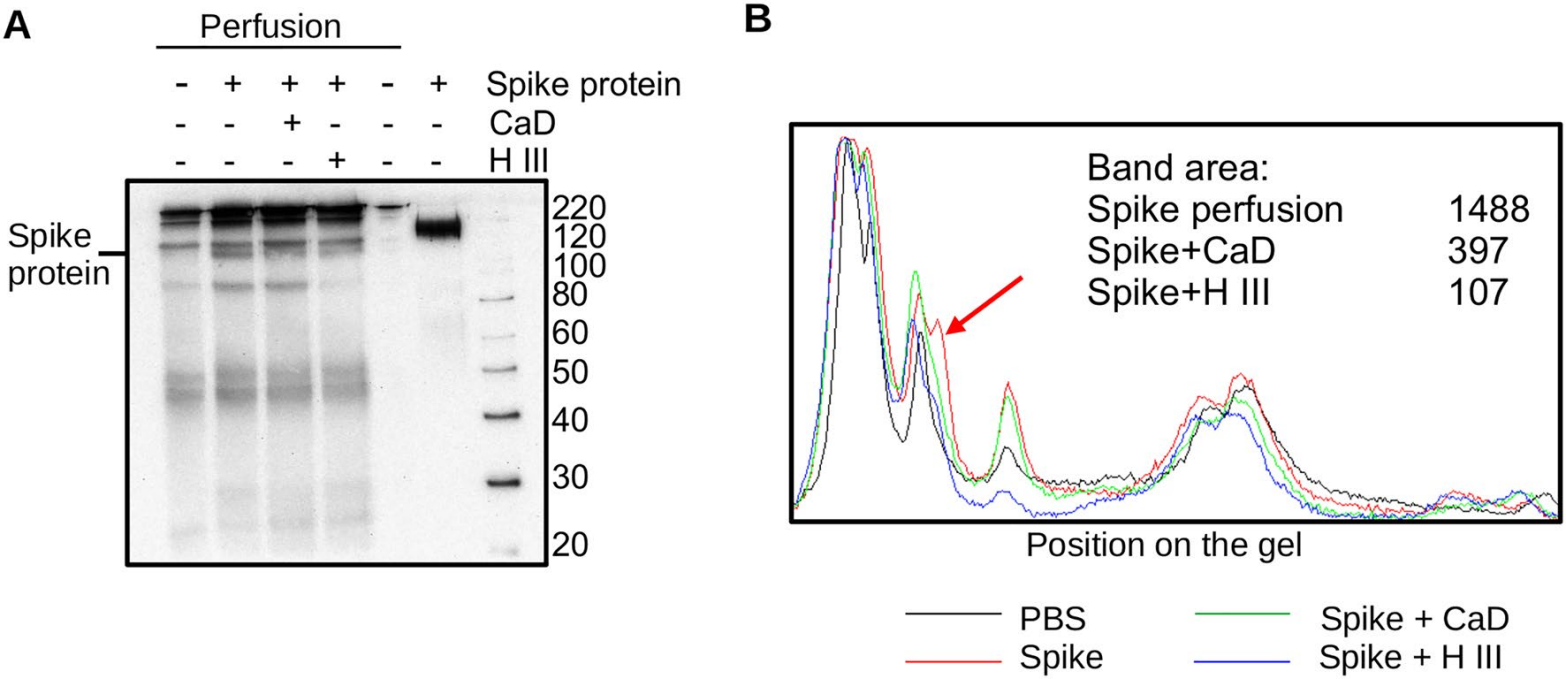
CaD prevents SARS-CoV-2 spike protein retention in ex vivo perfused mouse kidney. (**A**) Ex vivo perfused mouse kidney were homogenized in RIPA buffer in 1:10 ratio (W/W). Homogenates cleared by centrifugation were used for affinity precipitation of His-tagged spike protein using His-Mag Sepharose beads. Proteins were detected after western blotting using anti-His antibody. Recombinant spike protein used for the perfusion was used as a positive control. Position of molecular weight markers is shown on the right side. (**B**) Densitometry of the image shown in A was performed using QuantityOne software. Graphs show position on the image from the top to the bottom. Peak corresponding to the spike protein is marked with an arrow.

Perfused kidney tissues were homogenized and spike protein was affinity precipitated using His-Mag Sepharose beads. In western blotting we have observed that some amount of spike protein is retained in the kidney (Fig. 7A). This detainment was decreased by the addition of CaD and pre-treatment of the kidney with Heparinase III. Molecular weight of the spike protein bound to the kidney tissue differs from the positive control full length spike protein used for the tissue perfusion suggesting that proteolysis could take place. Figure 7B shows densitometry of the gel confirming decreased spike protein binding in the presence of CaD and after Heparinase III pre-treatment.

**Figure 8:**
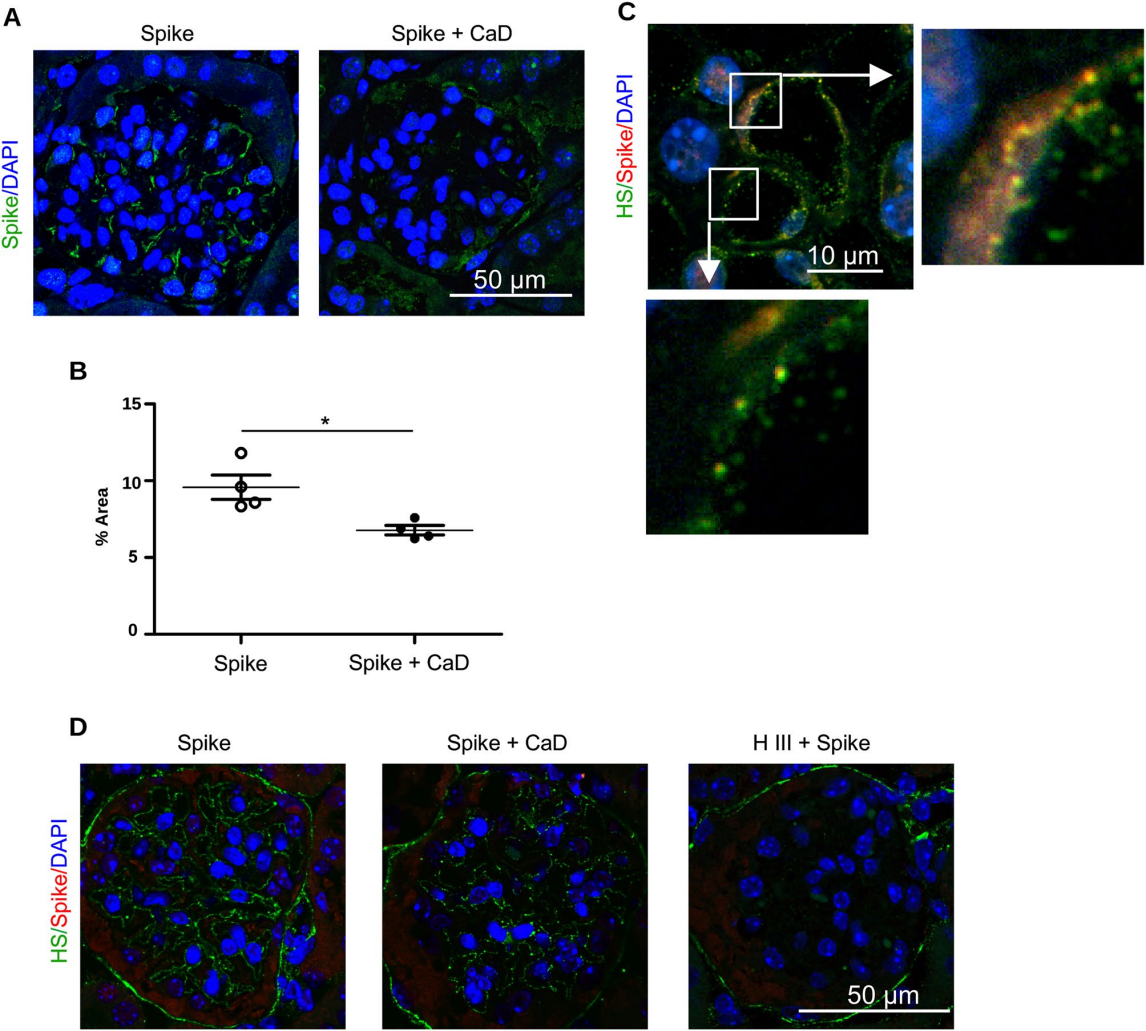
HS is essential for the retention of SARS-CoV-2 spike protein in ex vivo perfused mouse kidney. (**A**) Ex vivo perfused mouse kidney was stained using anti-spike protein antibody. Kidney glomerulus is shown. (**B**) Quantification of spike protein binding in kidney glomeruli using ImageJ software. (**C**) Peritubular capillary of the mouse kidney perfused with spike protein stained for HS (green), spike protein (red) and DAPI (blue). (**D**) Mouse kidney were perfused with spike protein pseudotyped lentivirus without or with 100 µM CaD. Kidney shown in right panel was perfused with heparinase III for 30 min prior the perfusion with spike protein.

Immunohistological staining using anti-spike antibody demonstrated the presence of spike protein on the surface of glomerular endothelial cells, (Fig. 8A). The amount of retained spike protein was significantly decreased in the presence of CaD (Fig. 8B). Figure 8C shows image of peritubular capillary. HS and spike protein partially co-localize on the luminal side of the capillary.

Next, we used spike protein pseudotyped lentivirus for the ex vivo mouse kidney perfusion. In accordance to the previous data, we observed some lentivirus detainment in the kidney (Fig. 8D) that was decreased by CaD and after heparinase III pre-treatment.

Together our data suggest that spike protein interacts with intact HS glycocalyx and will be retained in the tissues thus facilitating cell infection. CaD prevents spike interaction with HS and can protect the cells from being infected.

## Discussion

Using SARS-CoV-2 spike protein pseudotyped lentivirus particles and recombinant spike protein we demonstrate that CaD interferes with the initial process of spike protein binding to the HS on the cell surface and can represent a vital medicine to prevent SARS-CoV-2 infection.

Virus entry in a cell is a multistep process. Initial stages include low affinity interaction with abundant cell surface (co)receptor to increase local virus concentration and facilitate the search for higher affinity protein receptor for cell entry. Recent reports showed that various viruses use HS as the initial cell attachment site^24–30^. HS is ubiquitously expressed and present on the surface of nearly all animal cells and in the extracellular matrix. The overall high negative charge of HS is due to the presence of sulfate groups. The specific spatial arrangement of sulfate groups in short segments creates binding sites for protein ligands. Furthermore, HS has a very labile structure and demonstrates cell- and tissue-specific composition^54^⁠. Shedding of HS from the cell surface is also a fine tuned process observed in many pathological situations^55^⁠.

Endothelial cells develop especially pronounced glycocalyx on the luminal side. We have recently demonstrated that endothelial glycocalyx thickness decreases in covid-19 critically ill patients and serum of covid-19 patients induces shedding of endothelial cell glycocalyx in vitro^56^. It is still not well understood how different spatial compositions of HS, its occupancy by ligands (such as ECM proteins, growth factors) and shedding can affect virus attachment and cell infection in vivo. Increased plasma content of soluble HS fragments and proteoglycans can bind viral particles and decrease the probability of finding and interaction with cell surface receptors^57^⁠.

Inhibitory effects of heparin and other HS-mimetics on the cell infection by different viral types are known and the strategy was confirmed to be efficient against experimental SARS-CoV-2 infection^28,35–37^⁠. CaD has many advantages over heparin and HS mimetics. It is an approved vasoprotective drug without the adverse effects of, for example, increased coagulation; it can be applied as a nasal spray to prevent upper airways infections, or systemically through the oral route. Moreover, a recent case report of covid-19 demonstrated improvement on clinical parameters in a patient with covid-19 after CaD application^58^⁠.

Our in vitro binding assay confirmed that CaD can interfere with spike protein binding to HS. The kinetics were similar in the case of high and low molecular weight heparin. Binding of spike protein to HS was less efficient suggesting that sulfation is important for the interaction. However, we cannot exclude that lower binding was observed due to less efficient plate coating with HS.

Taken together, these data inferns that CaD can be potentially used to prevent cell infection with SARS-CoV-2 and urge further research towards clinical testing of this substance for the therapy of covid-19 patients.

Using different settings we demonstrated that CaD interferes with the interaction of spike protein with cell surface HS and virus infection. We observed CaD inhibition of spike protein binding using cell based ELISA. Interestingly, CaD increased binding of SARS-CoV-2 nucleocapsid protein to the cells. It is reasonable to assume that mechanism of CaD action on SARS-CoV-2 nucleocapsid protein is similar and implies protein interaction with heparan sulfate. To the best of our knowledge, there is no data available regarding the interaction of the SARS-CoV-2 nucleocapsid protein with heparan sulfate. Since heparan sulfate can also be found intracellularly and multiple intracellular proteins are capable of heparan sulfate binding such interaction may take place at the later stages of cell infection with SARS-CoV-2 and, most probably, not relevant to the initial stage of virus attachment to the cell surface. To test the hypothesis that nucleocapsid protein can interact with heparan sulfate we used the ClusPro computational prediction tool using heparin as a ligand^59–62^⁠. Several models of possible interactions has been proposed by the model confirming our hypothesis (Supplementary Fig. 3).

Further, we applied SARS-CoV-2 pseudotyped lentiviruses with secreted Gaussia luciferase and GFP as reporters. CaD interfered with HS dependent cell infection in the range of MOI from 1 to 50. CaD was effective under standard conditions of endothelial cell culture and under more physiological medium flow conditions during cell culture in microfluidic chips. Effects of CaD were generally abolished by enzymatic removal of HS from the cell surface using Heparinase III treatment.

We also addressed the issue raised in recent reports indicating that spike protein by itself can induce activation of inflammatory reactions^63,64^⁠. We used monocytic THP-1 cells, several types of endothelial cells, and HEK-Blue TLR4 reporter cell line stably expressing TLR4, CD14, and MD2 proteins. Our data are in agreement with the report of Ouyang and colleagues^65^⁠: we have not observed any direct inflammatory responses of spike protein (Supplementary Fig. 2B,C). Correspondingly, CaD had not demonstrated any effects in those experimental settings.

Rhea and colleagues have recently demonstrated that injected spike S1 protein can accumulate in various organs, including kidney, of wild type mice^66^⁠. Using ex vivo mouse kidney perfusion we confirmed that spike protein and spike protein pseudotyped lentiviral particles are retained in the kidney in a HS-dependent manner and CaD can prevent this. Our experiments focused on the uptake of the virus in endothelial cells. There is currently controversial data available whether endothelial cells express SARS-CoV-2 receptor ACE2 and/or other potential receptors and can be infected directly^19,20^⁠. Both, HMEC-1 and HUVECs expressed ACE2 and TMPRSS2 in our hands. Furthermore, data demonstrating the role of HS in virus infection and our data presented in this study point out that the situation is complex, and effects of glycocalyx has to be taken into account and investigated using more physiological models than standard cell culture. The role of glycocalyx in the receptor-independent endocytosis has been demonstrated for lipoproteins^67^. The size of lipoprotein particles is comparable with the size of viral particles and similar mechanisms of SARS-CoV-2 entry should be considered.

It is most likely that CaD also interferes with pseudo particle uptake in bronchopulmonary cells. These experimental studies are ongoing and may be the basis of a preventive treatment strategy using CaD. However, we believe that CaD preventing endothelial cell infection may be more crucial. We can envision a therapeutic strategy whereby patients in the early phases of SARS-CoV-2 infection are treated with CaD to prevent further spreading into the microvasculature. Such treatment could confine the infection to the bronchial tree and the patients would not suffer from the possible deleterious complications of microvascular disease. It is also possible that such a treatment strategy would prevent the chronic forms of the infection.

## Methods

### Materials and cells

Recombinant SARS-CoV-2 spike S1 subunit (40589-V08B1) was purchased from (Sino Biological), full length spike protein (10549-CV) and nucleocapsid (10474-CV) proteins were purchased from R&D Systems. Antibody against spike protein (NBP2-41058) was from Novus Biologicals; antibody against spike S1 subunit was from Sino Biological (40150-R007); HS antibody (10E4) was from Amsbio. Luciferase assay was performed using Pierce Gaussia Luciferase Glow Assay Kit (ThermoFisher Scientific, Waltham, MA, USA). CaD was purchased from Sigma (Merck KGaA, Darmstadt, Germany). Heparin, Heparan sulfate, LMW heparin Enoxaparin, Heparinase III were from Sigma. Toxicity of CaD was assessed using Cell Counting Kit-8 (Sigma, Merck KGaA, Darmstadt, Germany). Cell proliferation was measured by using BrdU incorporation assay (Roche Molecular Systems, Inc.). The effect of CaD on pinocytosis was assessed using FITC-Dextran (40,000 kDa, Sigma, Merck KGaA, Darmstadt, Germany). Effect of CaD on phagocytosis was measured using FITC-labelled E.coli as described recently^68^⁠. Fluorescence was measured using TECAN ELISA reader with Magellan software (Version 3.0x; https://www.tecan.com/). The following settings were used: excitation wavelength filter was 485 nm, emission wavelength filter was 535 nm. Integration time was 1000 ms. Duolink proximity ligation assay kit was purchased from Sigma (Merck KGaA, Darmstadt, Germany) and used accordingly to the manufacturer’s instructions. CY3-conjugated Sambucus Nigra Lectin (SNA, EBL) (CL-1303-1) was from Vector Laboratories, Inc. (Burlingame, CA, USA).

Qiagen RNAeasy kit was used for RNA purification from the cells. TaqMan RT-PCR was performed using TaqMan Master Mix and Light Cycler96 (Roche). TaqMan assays oligonucleotides were from ThermoFisher Scientific, Waltham, MA, USA.

Human dermal microvascular endothelial cells HMEC-1 were acquired from ATCC and cultured as recommended by the supplier. HUVECs were purchased from Lonza and cultured as recommended. THP-1 cells were from ATCC. HEK-Blue-hTLR4 were from Invivogene.

Chemical structure of CaD was drawn using MolView online tool (https://molview.org/).

### Preparation of vectors for lentivirus based secreted Gaussia luciferase (GLuc) and GFP expression

cDNA of GAPDH promoter and Gaussia Luciferase (GLuc) were derived from pEZX-LvPG04 vector (GeneCopoeia, Inc., Rockville, MD, USA) used as a template. A full-length cDNA of GAPDH promoter and GLuc were amplified using Phire Hot Start II DNA Polymerase (Thermo Fisher Scientific, MA, USA). The sequences of all primers used are given in Supplementary Table 1. PCR products were gel purified by QIAquick PCR Purification Kit (Qiagen, Hilden, Germany). Promoterless Gaussia luciferase lentivirus vector was generated by In-Fusion cloning kit (TaKaRa Bio, CA, USA). HPRM30418-LvPG04_pEZX-LvPG04 (GeneCopoeia) was used as lentivirus backbone. Vector was linearized by EcoRI and XhoI (ThermoFisher Scientific, Waltham, MA, USA) and circularized in In-Fusion reaction with different PCR products. GAPDH promoter was inserted in the promoterless pEZX-GLuc in MssI (PmeI) (Thermo Fisher Scientific, Waltham, MA, USA) site in this manner. In order to obtain fluorescent versions of constructs, cDNA of GLuc was replaced on cDNA TaqGFP protein. Plasmid pTagGFP-C (Evrogen, Moscow, Russia) used as template for PCR reaction. PCR primers and target DNA described in Supplementary Table 1. The pEZX vector encoding GLuc and GAPDH promoter were transformed into an Escherichia coli strain (*E. coli* DH5α). The products were verified by sequencing (Microsynth Seqlab, Göttingen, Germany).

To generate pseudotyped viral particles with the SARS-CoV-2 spike protein on a lentiviral core, spike glycoprotein expression plasmid pLV-Spike V1 (SARS-CoV-2 Wuhan-Hu-1 isolate with the D614G mutation) was used (https://www.invivogen.com/wuhan-spike-d614g-pseudotyping-vector). Second-generation lentiviral packaging construct plasmid psPAX2 (expressing HIV-gag) was acquired from Addgene (http://www.addgene.org/12260/). The work was performed under S2 biosafety level. Pseudoviral particles were produced by transforming packaging cell line HEK293T using Perfectin transfection reagent from Genlantis (San Diego, CA, USA) as established^69^. HEK293T conditioned medium containing lentiviral particles was centrifuged at low speed, filtered through 45 µm pore size filter, and stored at + 4° C for up to 1 week before cell infection. Expression of spike protein of SARS-CoV-2 in lentiviral pseudoparticles was confirmed by Western blotting using SARS-CoV-2 (COVID-19) spike antibody [1A9], IgG1 from Genetex (GTX-GTX632604, Irvine, CA, USA). Lentivirus titer was determined using Quick Titer kit from Cell Biolabs (Cell Biolabs, Inc., San Diego, CA, USA). Lentivirus was added at the MOI 50 for 2 h where not indicated otherwise. For infection, inhibitors were added to the lentivirus containing supernatant, and the cocktail was added to the cells for 2 h. After the incubation, the medium containing the remaining lentivirus was removed, cells were washed once with a warm growth medium, a fresh medium was added and cells were incubated for 24 h before measurement of Gluc activity or microscopy.

Gaussia luciferase activity was measured using Pierce™ Gaussia Luciferase Glow Assay Kit (Thermofisher Scientific).

### Spike protein binding to heparin, HS, and LMW heparin-coated plates

High binding ELISA plates were coated with heparin, enoxaparin and HS as described by Clausen et al.^33^⁠. Plates were blocked with 0.1% BSA on PBS, washed and then recombinant His-tagged spike protein (R&D Systems) was added at the concentration of 30 nM along with the indicated concentrations of CaD. Spike protein detection was performed using anti-His antibody (R&D Systems).

### Microfluidic experiments

We applied developed by us microfluidic system described elsewhere^53,69^. Briefly, cells were seeded in the channels of polydimethylsiloxane (PDMS) microfluidic chips and allowed to adhere for 6 h. Then, medium perfusion was initiated using peristaltic pump (Watson Marlow) and teflon tubing (Diba Industries). Cells were perfused with shear stress of 16 dyn/cm^2^ for 5 days before further experiments. To exclude absorption of lentiviruses to the teflon tubing and PDMS, control experiments were performed. Lentivirus containing medium was perfused through the tubing and empty microfluidic chips. Then, this medium was used for cell infection and virus quantification. These experiments showed that there were no absorption of the lentivirus to chips and tubes.

### Western blotting

Western blotting was performed as described previously^53^. Briefly, after electrophoresis the proteins were transferred to PVDF membrane using semi-dry western blotting apparatus (Bio-Rad Laboratories GmbH, Feldkirchen, Germany). Chemiluminescence pictures were taken using VersaDoc gel documentation system and quantified by QuantityOne software (Version 4; https://www.bio-rad.com/de-de/product/quantity-one-1-d-analysis-software?ID=1de9eb3a-1eb5-4edb-82d2-68b91bf360fb).

### Cell staining and microscopy

Cells were fixed with 2% PFA and blocked with 1% of BSA on PBS. Duolink assay was performed using kits from Sigma following the protocol suggested by the company. Confocal microscopy was then performed using Leica TCS-SP2 AOBS confocal microscope (Leica Microsystems) at the Research Core Unit for Laser Microscopy at Hannover Medical School. Quantification of the images was performed using ImageJ analysis software (ImageJ 1.48v; https://imagej.nih.gov/ij/).

### Ex vivo mouse kidney perfusion

Ex vivo experiment was performed with kidneys obtained from healthy C57BL6N wild type mice (12 weeks of age, 23–25 g bodyweight). Animals were euthanized humanely accordingly to Paragraph 7, Section 3 of German Animal Welfare Act. All experiments were approved by the animal protection committee of the Phenos GmbH (Tierschutzkomission der Phenos GmbH). All animals were handled in compliance with the guidelines for the care and use of animals at our institution and in accordance with the EU Directive 2010/63/EU for animal experiments. Methods are reported in accordance with ARRIVE guidelines. C57BL6N wild type male mice were purchased from Charles River Laboratories (Sulzfeld, Germany). The following groups were used with three animals per group: PBS perfusion; spike protein perfusion; spike protein with CaD perfusion. The mice were euthanized humanly and both kidneys, abdominal aorta and inferior vena cava were exposed via abdominal incision. Small vessels were cauterized with the electrosurgical pencil and cut. Proximal ends of the aorta and vena cava and the distal aorta and vena cava near bifurcation were also clotted and cut. Ureters were cut to separate both kidneys. A 7/0 ligature was preset around the aorta. The preset ligature was closed after inserting 27G catheter without core into the aorta. Kidneys were perfused with sterile saline to remove the blood.

Both kidneys with catheter were moved to a dry dish, the catheter was connected to the peristaltic pump (Watson Marlow) and perfusion of the kidneys with saline was continued for 5 min. Based on reported blood flow rate in the mouse kidney^70^, perfusion rate was set to 1.3 ml/min. Kidneys were perfused with PBS (Group 1); 15 µg/ml spike protein (Group 2); 15 µg/ml spike protein with 100 µM CaD (Group 3) for 30 min. Where indicated, kidneys were perfused with heparinase III 50 mU/ml for 30 min prior perfusion with spike protein or lentiviruses. Fluid flowing from the vein was absorbed back into the perfusion solution tube by another catheter connected to the peristaltic pump. Then, the kidneys were perfused with saline for 5 min, washing solution was collected in 1 ml fractions for western blotting analysis. After perfusion kidneys were cut into three parts; upper and lower parts were frozen in liquid nitrogen for biochemical experiments, the middle part was fixed in formalin for immunohistochemical staining. For His Mag sepharose pull down assay, the tissue were homogenated in 10:1 (v:w) ratio in RIPA lysis buffer and incubated for 15 min on ice. Lysates were cleared by centrifugation and used for the pull down assay.

### Statistics

All data were obtained with at least three biological replications. Data are presented as mean ± standard deviation (SD). Multiple comparisons were analyzed by ANOVA with Tukey post hoc test. P-values < 0.05 were considered statistically significant. GraphPad Prism 8.3.0 (https://www.graphpad.com/) was used for data analysis.

### Supplementary Information

Supplementary Information.

## Data Availability

All data generated or analysed during this study are included in this published article.
