# Author Correction: Calcium dobesilate reduces SARS-CoV-2 entry into endothelial cells by inhibiting virus binding to heparan sulfate

**DOI:** 10.1038/s41598-023-44504-w

**Published:** 2023-10-13

**Authors:** Yulia Kiyan, Anna Schultalbers, Ekaterina Chernobrivaia, Sergey Tkachuk, Song Rong, Nelli Shushakova, Hermann Haller

**Affiliations:** 1 Department of Nephrology, Hannover Medical School, Carl‑Neuberg‑Str. 1, 30625 Hannover, Germany; 2 Mount Desert Biological Laboratory MDIBL, Bar Harbor, USA; 3 Phenos GmbH, Hannover, Germany

Correction to: *Scientifc Reports* 10.1038/s41598-022-20973-3, published online 07 October 2022

The original version of this Article contained an error in Figure 3, panel B, where one of the images was incorrectly displayed.

**Figure 3:**
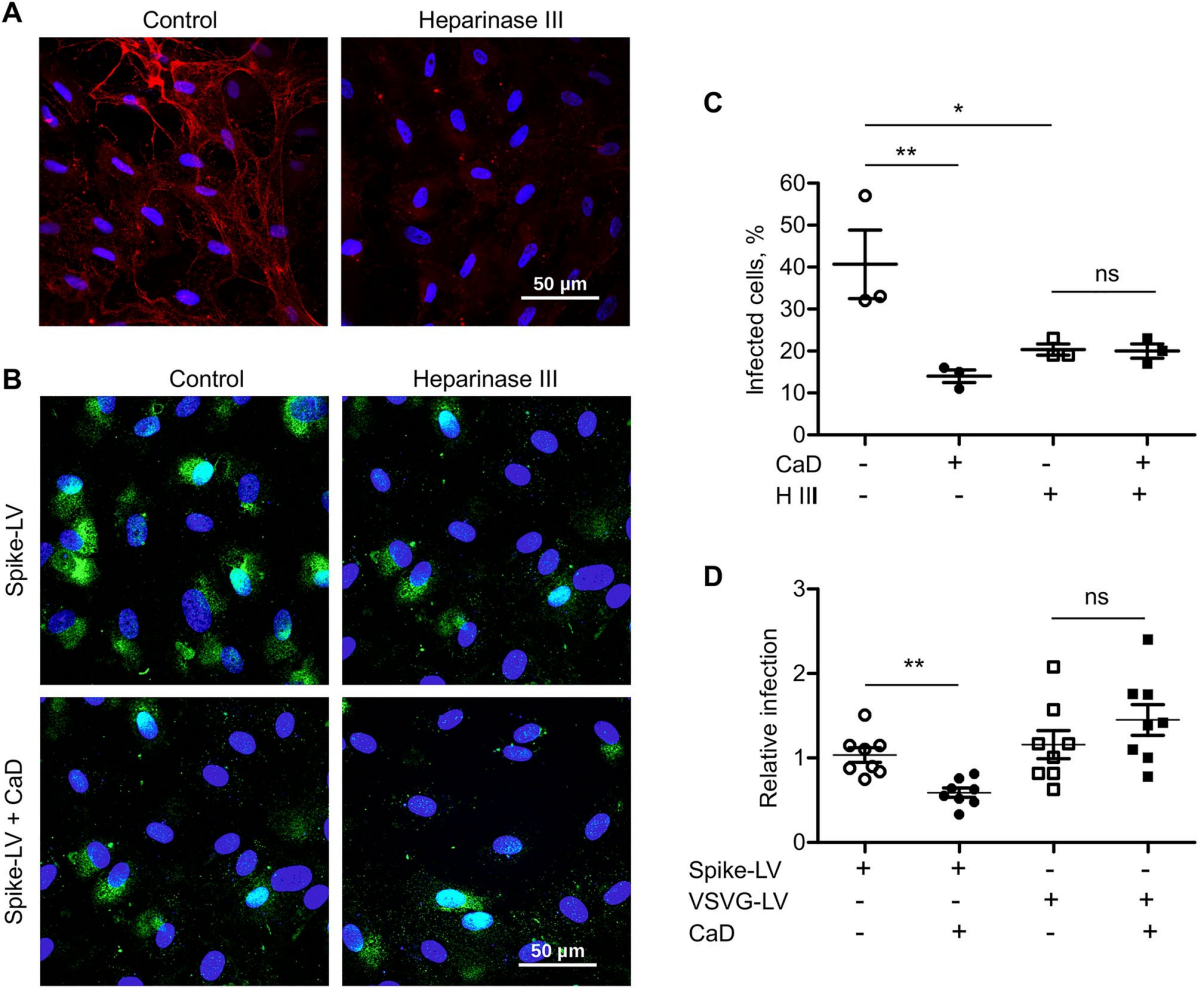
CaD inhibits interaction of SARS-CoV-2 spike protein with HS. (**A**) HUVECs were treated with Heparinase III for 1 h and then fixed and stained with heparan sulfate antibody (red) and DAPI. Intracellular HS staining is still present, whereas extracellular fibrous HS staining was removed by Heparinase III treatment. (**B**) Heparinase III treatment was performed for 1 h at 37 °C prior cell infection. HUVECs were infected with spike-pseudotyped lentivirus (Spike-LV) expressing GFP as a reporter in the presence or absence of 10 µM CaD. 24 h after infection cells were fixed and expression of GFP was visualized by microscopy. (**C**) The number of pseudovirus-infected GFP positive cells was Quantified using ImageJ. (**D**) TaqMan RT-PCR was performed to quantify GFP mRNA expression in HUVEC infected with Spike-pseudovirus and VSVG protein lentivirus (VSVG-LV) in the presence of CaD.

The original Figure 3 and its accompanying legend appear below.

The original Article has been corrected.

