# Supplementary material for: Calcium dobesilate reduces SARS-CoV-2 entry into endothelial cells by inhibiting virus binding to heparan sulfate
Source: Sci Rep. 2022 Oct 7;12:16878. doi: 10.1038/s41598-022-20973-3 (PMC9542452; doi:10.1038/s41598-022-20973-3)
Supplement: Supplementary file 1 — Supplementary Information. [file 41598_2022_20973_MOESM1_ESM.pdf]

Supplementary information

Calcium dobesilate reduces SARS-CoV-2 entry into endothelial cells by inhibiting virus binding to heparan sulfate

Yulia Kiyan, Anna Schultalbers, Ekaterina Chernobrivaia,

Sergey Tkachuk, Song Rong, Nelli Shushakova, Hermann Haller

Supplementary Table 1.

Gene	Primer sequence	
GLuc	D	5´-CCAGATCTTGGAATTGTTTAAACATGGGAGTCAAAGTTCTGTTTGCCCTG
	R	5´-CATTCCACAGCTCGACTAGTCACCACCGGCCCCC
GAPDH	D	5´-ATCTTGGAATTGTTTCGGGATTGTCTGCCCTAATTATCAGG
	R	5´-TTGACTCCCATGTTTGGAGAGAACAGTGAGCGCCTAGTG
TagGFP	D	5´-CCAGATCTTGGAATTAATTAATGAGCGGGGGCGAGGAGC
	R	5´-CATTCCACAGCTCGATTATCTAGATCCGGTGGATCCCGG

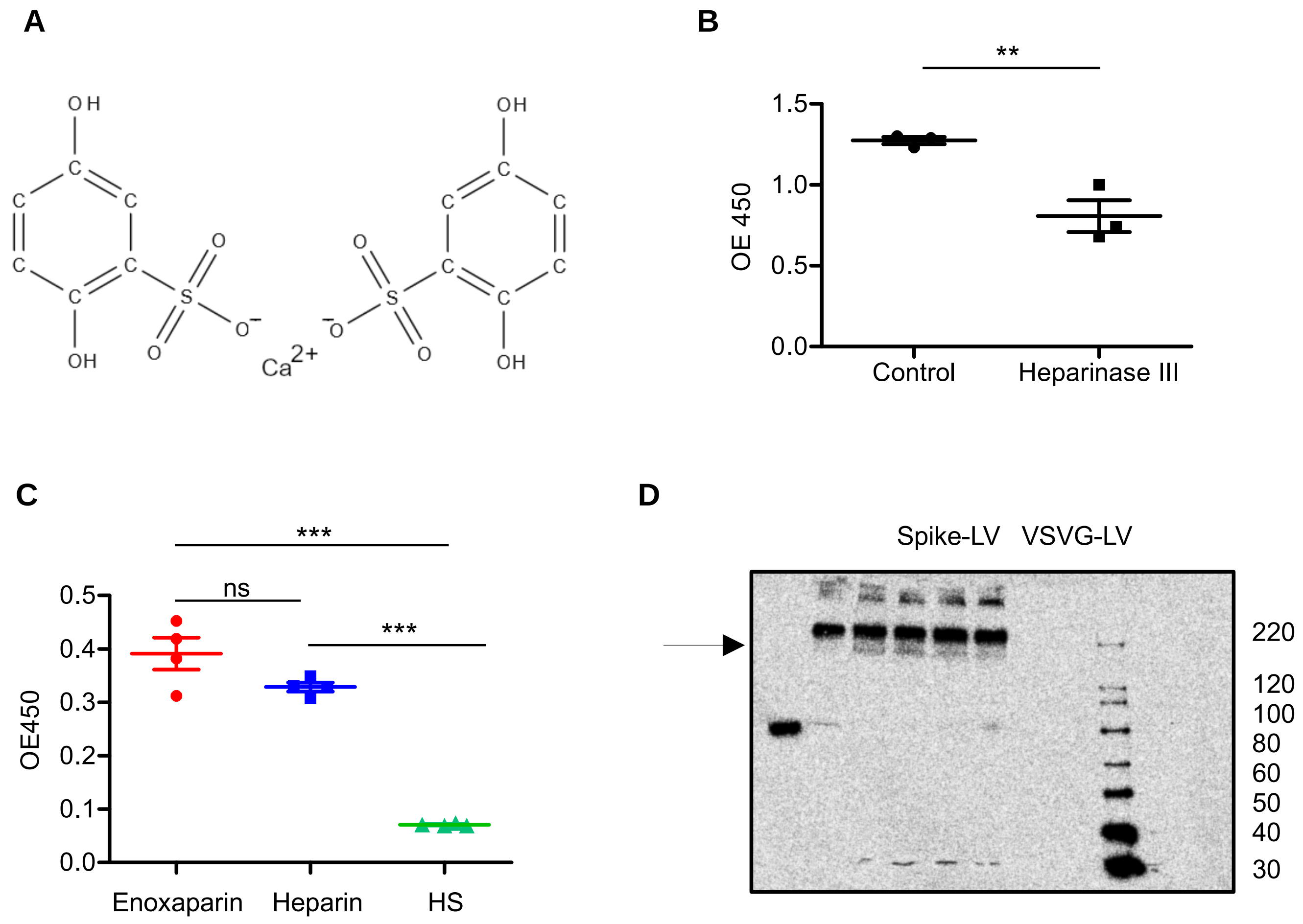

Supplementary Figure 1. A. Chemical structure of CaD. B. Cell-based ELISA staining using HS antibody. C. Binding of recombinant spike protein to the plates coated with enoxaparin, heparin and HS detected using anti-His antibody. D. Spike protein detection in the pseudotyped lentivirus preparation.

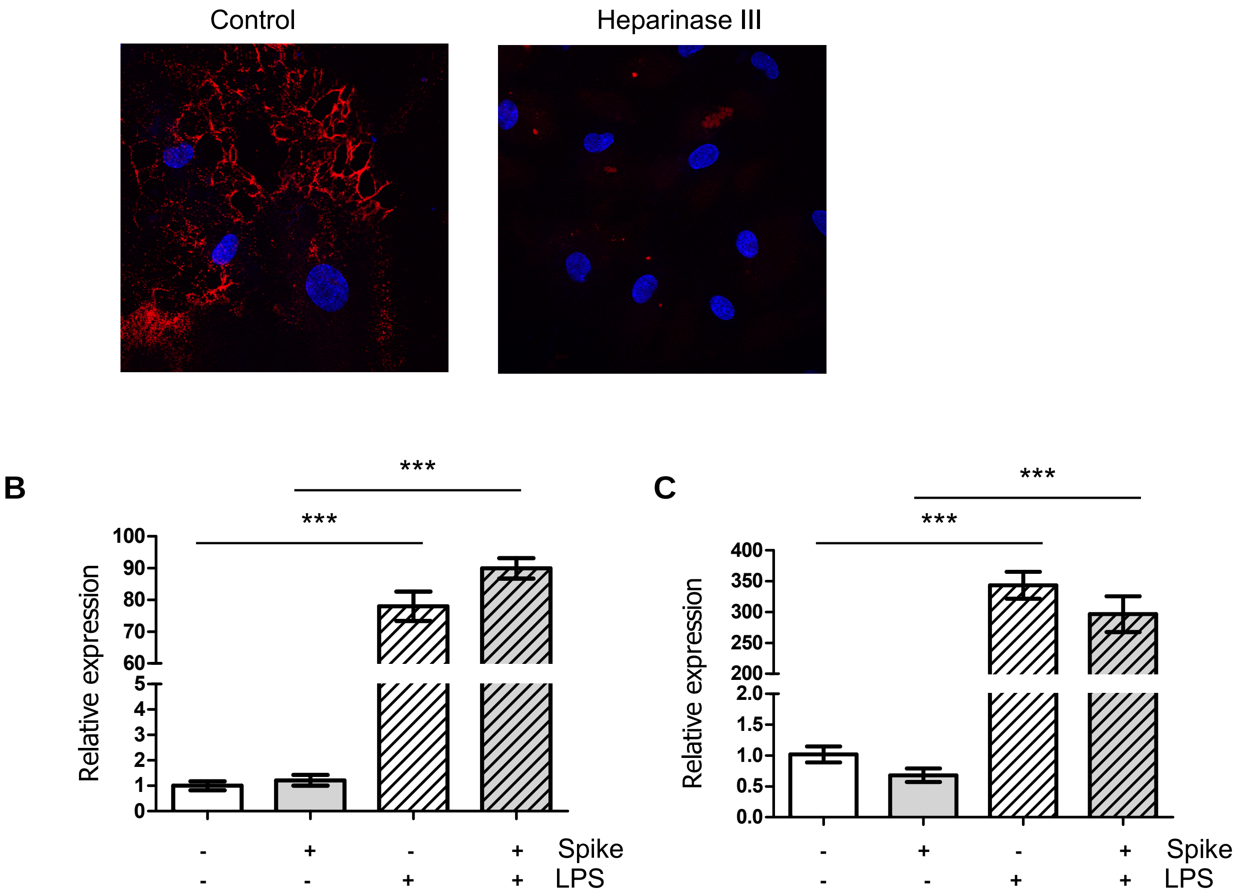

Supplementary Fig. 2. A. Control HS staining of the cells used for Duolink assay. B. IL-6 expression assessed by RT-PCR after 3 hours of cell stimulation with 500 ng/ml of Spike protein in the absence or presence of 10ng/ml LPS. C. MCP-1 expression assessed by RT-PCR after 3 hours of cell stimulation with 500 ng/ml of Spike protein in the absence or presence of 10 ng/ml LPS.

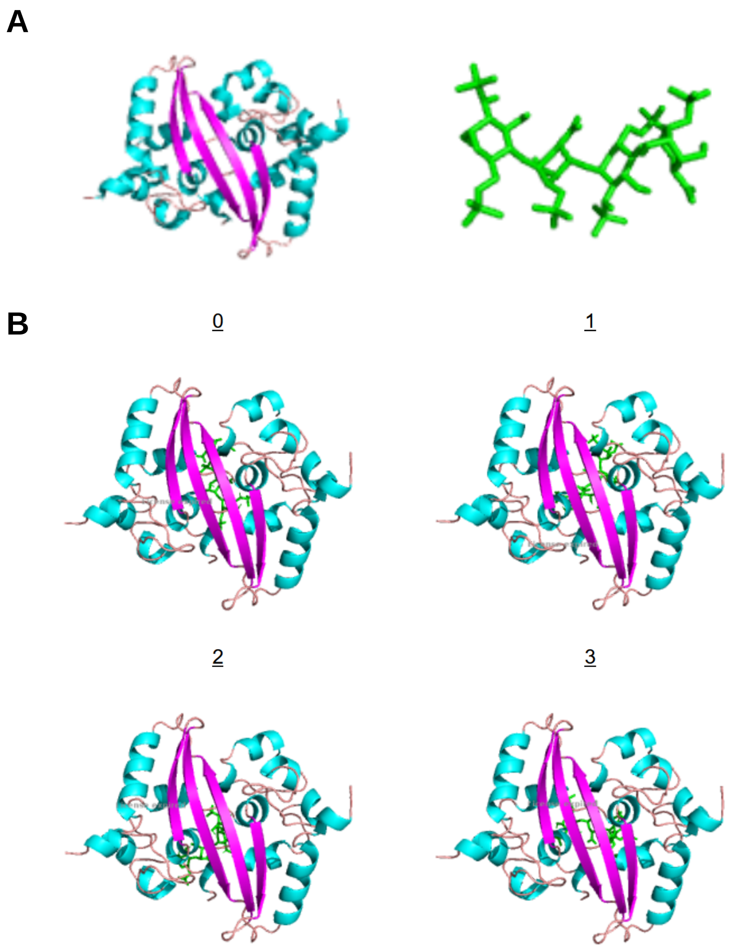

Supplementary Figure 3. Modeling SARS-CoV-2 nucleocapsid protein interaction with heparin using ClusPro protein protein docking tool. A. Structure of SARS-CoV-2 nucleocapsid protein C-terminal domain (left) and heparin (right). B. High rank models of SARS-CoV-2 nucleocapsid protein interaction with heparin.

A

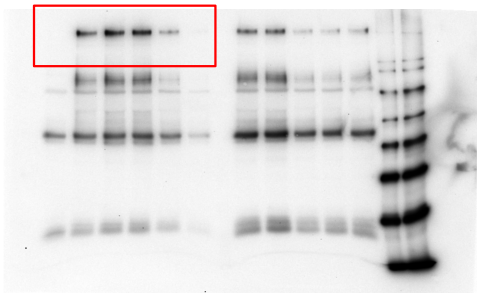

B

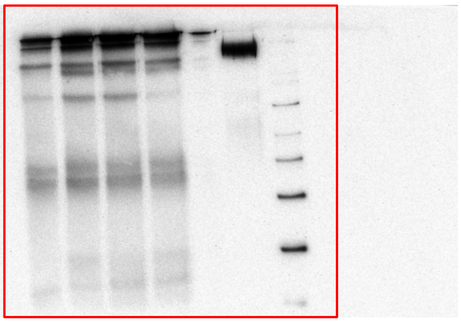

Supplementary Figure 4. Full size gels for Fig. 6 (A) and 7 (B). Are shown in the corresponding figure is marked by red rectangle.
